# SMES survival and knowledge in emerging economies: evidence from Vietnam

**DOI:** 10.1016/j.heliyon.2022.e11387

**Published:** 2022-11-04

**Authors:** Nguyet Nguyen Thi

**Affiliations:** Vietnam Institute for Development Strategies, Ministry of Planning and Investment (MPI), Viet Nam

**Keywords:** Knowledge, SMEs, Firm survival, Cox model, Emerging economies

## Abstract

This paper evaluates how knowledge affecting the survival of new private small and medium sized enterprises (SMEs) in an emerging economy like Vietnam. The panel data extracted from the national surveys allow for comprehensive analysis during the period 2005–2011 with the semi-parametric Cox proportional hazard model. The study examines knowledge factors under controlling firm-specific and industrial factors. The findings express strong evidence that Vietnamese SMEs benefit strongly from knowledge development. The findings suggest firms to foster labor productivity, value added, and computerization. There is evidence of market selection, based on efficiency of investment. Besides, firms should improve return on sales, especially in growing market. Start-up SMEs should prepare sufficient total assets, not basing on leverage to enter the market. Start-up in industries with high growth, especially at the early stages of the life cycle will require more government support. These findings provide the evidence for policy improvement to help SMEs in the development of knowledge economy.

## 1 Introduction

Knowledge economy (KE) is “based on the view that information and knowledge are at the center of economic growth and development” (OECD, 2001, p.99). Promoting development of KE in Vietnam will contribute to ensure the quality of growth, efficiency, and competitiveness of the economy (Vu and Nguyen, 2014). This study indicates that the current economic policies in Vietnam are reducing the effectiveness of technology application then hindering enterprises, especially Small and medium-sized enterprises (SMEs), from developing.

Private SMEs not only have the most potential role for stable growth (Nguyen and Ngo, 2021) but also the most important pilar for Vietnam's economic development recently. Since Vietnam's Reform, with the desire to gradually eliminate the subsidized bureaucratic mechanism of the planned economy and transiting towards market economy, Vietnam has prepared and promulgate policies for the development of private enterprises. Recently, the private SME sector, accounting for about 97% of total enterprises (GSO, 2013, 2021), has become the main growth engine of the Vietnamese economy.

Nevertheless, the failure rate of firms in Vietnam has extraordinarily increased during 2005–2011, especially in 2011 the exiting rate of firms was more than 12%, four-fold the normal rate due to facing some difficulties (GSO, 2013). In 2011, the extremely high lending interest rates which were pushed to 24–25%, and CPI in December 2011 compared to 2010 increased by 18.58%, reaching the highest level in Asia (GSO, 2011), causing many difficulties for businesses. In addition, the contribution of total productivity factor (TFP) to growth in the 2006–2010 reached 17.2%, was the lowest in the period 2001–2015 (21.4% for the period 2001–2005; 28.94% for the period 2011–2015) (Mai, 2018).

The research significance comprises: (i) The study examines a comprehensive set of knowledge as well as control variables at firm and industrial levels to evaluate the effects of the knowledge on the survival of Vietnamese SMEs; ii) and compare their changes under different contexts, among different firm sizes and locations; iii) The paper investigates the effects of market selection, Start-up factors on firm survival which findings could provide the evidence for policy improvement to help SMEs in the development of knowledge economy.

The novelty of the study includes: (i) it gives bases for the future policy renovation to support Vietnamese enterprises because determinants of firm survival are important to understand the selection market (Esteve-Pérez and Máñez Castillejo, 2008); (ii) The panel data are extracted from the national statistical surveys with rich and representative information which allow for in-depth analysis; iii) the Cox proportional hazard model has developed progressively to give more efficient estimations, resolving challenges such as discrete time, unobserved heterogeneity, and competing risks.

The research concentrates on the following hypotheses:

(i) The knowledge improves the survival of Vietnamese private SMEs
(ii) The effects of the knowledge on Vietnamese private SMEs'*survival* depend on internal and external factors
(iii) The effects of knowledge on Vietnamese private SMEs'*survival* depend on firm size and location.

After the introduction, the research includes: section 2 expresses the employed methodology with model, variables, and data. section 3 presents the empirical results and analysis while the final one presents conclusion and policy implications.

## 2 Literature review

### 2.1 Firm survival

For knowledge factors, the number of computers is investigated because ‘computers are best described as a general-purpose technology’ (Brynjolfsson and Hitt, 2003, pp. 793). Besides, knowledge economy presents significantly “greater share of both value-added and employment in the business sector” (OECD, 2001, p. 101), thus value added is researched. In addition, there is close relationship between patents, R&D or human capital with firm productivity (Ghosa and Nair-Reichert, 2009), higer productivity will ensure higher probability of firm survival (Baily et al., 1992; Doms et al., 1995; Triplett, 1999; Esteve-Pérez–Máñez Castillejo 2008). Moreover, the decisive factor increase Long-term growth is the strength of capital and technology (CIEM, 2013).

Regarding *Profitability factors*, Mahmood and Mann (1993) said that there is a strong relationship between information technology, presented for knowledge development, and profitability which could be presented by return on investment and return on sales.

In terms of sustainablity, Leverage and Debt per Owner Equity are proxies for firm sustainability (Nguyen, 2016). With respect to s*tart-up factors,* start-up total assets and Start-up Leverage are found as important determinants of firm survival (Dunne and Hughes, 1994; and Jensen et al., 2008).

With respect to *Industrial factors,* market concentration and the market share are tested for the statement that the impact of science and technology investment on the firm performance depends on the industrial characteristics (Carpenter and Petersen, 2002; Nguyen, 2016).

In terms of methodology, similar to Manjón-Antolín–Arauzo-Carod (2008), “Firm failure” in this study means the firm disappearance from the market, then survivors are those appeared. The “Firm failure” function presents probability that a firm disappears at time t under the condition that it was survival until t (Stevenson, 2009). The hazard rate of firm failure presents the instantaneous rate that firm i disappears at time t under the condition of firm survived up to time t. This research applies the Cox proportional hazard model rather than the principal component analysis or Analytical Hierarchy Process because CPH is more advantaged than other approaches by handling well the three issues of survival time data which other could not, including: i) time-varying covariates; ii) censoring (and truncation); iii) structural modelling (Jenkins 2005). The advance of CPH is the non-parameterised baseline hazard, that means an assumption about the baseline hazard shape is not necessarily required (Blossfeld–Rohwer, 1995; Harris–Li 2010). Because the shape of the baseline hazard recently could not identify exactly due to insufficient theory, thus survival studies have to make a specific assumption and CPH model could control such characteristics.

The stochastic events of the interested variable can be described completely by hazard function (Manjón-Antolín–Arauzo-Carod. 2008). The survival time is the spell from the start-up time of a company to the disappearance time. In this study, spell is incomplete because the time period is not fully observed (after ending time, year 2011), that is right censoring (Manjón-Antolín and Arauzo-Carod, 2008).

### 2.2 Vietnamese SMEs

Vietnam had conducted the reform –“Doi moi” in 1986 with the transition to a "Socialist-oriented market economy" from a centrally planned economy. The year 1986 marked the first time private sector was recognized, then strongly developed with the privatization launched from 1992. The first Law on Enterprises was issued in 2000 along with a series of reform policies and guidelines of the Party to create a clear environment to support enterprises of all economic sectors, it opened a booming and fast-growing period for Vietnamese enterprises.

SMEs had played an important role in economic development, in the period of 2000–2020, contributing more than 40% of the country's GDP and 30% of the total state budget revenue on average. The SME sector has been a major contributor to job creation, poverty alleviation, improved living conditions and inclusive and sustainable development. SMEs created 5.98 million jobs in 2010 and this number increased to 8.8 million in 2018. In addition, the development of the corporate sector made an important contribution to the expansion of social insurance participants with the proportion of the labor force of age participating in social insurance increasing from 28.4% in 2017 to 32.5% in 2019 (Le and Tran, 2021).

SMEs developed rapidly in the whole period of 2000–2015 and the average growth rate in the period 2000–2015 was quite fast with 18.1%/year, while the large-scale enterprise sector only increased by an average of 7.3%/year (GSO, 2017). The size of enterprises in Vietnam was still mainly small and medium-sized enterprises (currently accounting for approximately 98% of all enterprises in terms of the number of employees, of which approximately 69% of enterprises have less than 10 employees). In general, productivity, quality and production and business efficiency of Vietnamese enterprises tend to decrease gradually, especially in the period 2010–2015 (affected by the global economic decline) much lower than the period 2000–2010. The smaller the enterprise, the lower the rate of profitable activities was and the business activities of small and micro enterprises faced more difficulties (VCCI and USAID, 2019).

The annual firm survival rate for 2013–2015 was 90.6%, down from the previous 2011–2013 survival rate of 91.3%. This was equivalent to the average withdrawal rate in developing countries of 9–10% (Liedholm and Mead, 1999). In 2019, only 54.1% of all registered SMEs actually operated, it was worth noting that the gap between the number of registered enterprises and those that were actually operating (or surviving) was widening (UNU-WIDER, 2016). By 2020, to withstand the pandemic, small and medium-sized private enterprises must lay off workers at the highest rate, at 36% and 35% respectively (Duong et al., 2022).

This shows a challenging business environment for most private enterprises in Vietnam. Vietnamese enterprises, especially micro and small enterprises, have slow development scale due to main limitations in the business environment, access to capital and finance, human resources, management skills, market access, technology and innovation capacity.

## 3 Methodology

### 3.1 Research model

The hazard function is given by:

(1) $$\lambda_{i}\left(t;X_{it}\right)=P\left[firm\;i\;at\;t|survival\;to\;t;\;X_{it}\right]=P\left[T=t|T\geq t,X_{it}\right]$$

where $\lambda_{i}\left(t\right)$ the hazard rate of firm failure.

With the hazard function in Eq. (1), following Cox (1972) the Cox proportional hazard (CPH) model has the following form:

(2) $$\lambda_{i}\left(t\right)=\lambda_{0}\left(t\right)\exp\;X_{it}\beta$$

where

$\lambda_{0}\left(t\right)$ is the nonparametric base-line hazard.

$X_{it}\beta$ expresses the parameterised function of explanatory variables.

$X_{it}$ is a matrix of knowledge factors, other internal and external factors which were assumed to influence the hazard rate.

β is a matrix of coefficients of factors.

This semi-parametric model is more advantaged than other models due to avoiding misspecification function of the baseline hazard (Harris and Li, 2010).

In this research, new SMEs are classified as firms born in 2005, and will be investigated during the period 2005–2011. The unit of time analysis is measured by years, named firm age.

To address unobserved factors, for examples management quality, prestige and fame (Manjón-Antolín–Arauzo-Carod, 2008), the CPH model in Eq. (2) can be developed as follows:

(3) $$\lambda_{i}\left(t\right)=\lambda_{0}\left(t\right)\exp\;X_{it}\beta *v_{i}=\lambda_{0}\left(t\right)\exp\;X_{it}\beta +u_{i}$$

where

$v_{i}$ characterises an individual heterogeneity that is unobserved.

$v_{i}$ is assumed to have unit mean, a gamma distribution and finite variance $\sigma^{2}$.

In Eq. (3), the null hypothesis of “no unobserved heterogeneity” will be tested by the option of shared frailty.

This paper applies the research model expressed in Eq. (3) to investigate how knowledge affecting the survival of new private small and medium sized enterprises for the case of Vietnam. The annual data employed in this study is discrete time, thus there are ‘ties’ in grouped-form data. This study applies approximation method constructed by Efron (1977) to address these ties.

### 3.2 Variables

As mentioned before, the dependent variable, hazard rate, is the probability of a firm exiting at year t under the condition that it was ‘alive’ until year t (Manjón-Antolín–Arauzo-Carod, 2008). The independent variables are theoretically driven as follows (see Table 1):

**Table 1:** Variables. Source: own analysis.

Independent variable	Explanations
Internal factors	
*Knowledge factors*	
Number of computers	Log of number of used computers
*Value added*	Log of value added
Labour productivity	Total sales divided by total number of employees.
Capital intensive	Total fixed asset per labour
*Profitability*	
Return on investment	Net income divided by its total investment.
Return on Sales	Net income divided by its total Sales.
*Sustainability*	
Leverage	Total liabilities divided by total assets (The book value)
Debt per Owner Equity	The ratio of total Debt per total Owner Equity
*Start-up factors*	
Start-up total assets	Total assets at the foundation year
Start-up Leverage	Total liabilities divided by total assets at the foundation year
***External factors***—*Industrial factors*	
Market concentration	Herfindahl–Hirschman Index (HHI): measured by squaring the market share of each firm in the market, and then summing that numbers
Market share	The share of each firm total sales per the total sales of whole industry

All financial variables in this paper are deflated with CPI—the annual consumer price index.

### 3.3 Data

This study employs data from the national enterprise survey in Vietnam from 2005 to 2011, which are conducted jointly by GSO (the General Statistical Office of Vietnam) and World Bank. This survey covers all firms in all sectors of Vietnam economy. Nevertheless, this paper only investigates the new private SMEs. The concept of ‘new’ private SMEs, refers to those that were born only in 2005 (without concerning to those born in the year different from 2005).

The data are employed for period 2005–2011 because this period has experienced an outstanding institutional reform for enterprises as well as knowledge development in Vietnam. The year 2005 has remarked the first time when Enterprise Law has unified regulations on the establishment and management of all types of enterprises, regardless of whether they are state-owned or privately owned, eliminating unreasonable differences between enterprises of different economic sectors, and expanding business freedom. Furthermore, this time has observed the strong reform in innovation and technology framework, operating the national scientific and technological tasks. In the period 2005–2011, Vietnam has experienced strong fluctuation in economic development. In the period 2006–2007, the average economic growth reached 8.34%, while only reached 6.14% for the period 2008–2010 due to the impact of high inflation and the world economic recession (GSO, 2011). Especially the year 2011 has witnessed the outstandingly high rate of firm failure (GSO, 2013). In addition, in the national survey on enterprises in Vietnam, the information of number of computers used in firms is annually available only for period 2005–2011 (this variable is not collected for later years). Therefore, this research focuses on evaluating how knowledge affecting new SMEs’ survival in a developing country like Vietnam for the period 2005–2011.

Besides, the study only considers Vietnamese wholly owned SMEs, the foreign partnership companies are not taken into account. The employed dataset is right censored, similar to most other empirical studies, because the survival firms cannot be observed after year 2011—the time ending in this study. The applied methodology has accounted for this right censoring. Besides, missing values are excluded then the applied dataset include 6.752 medium sized firms and 6.514 small sized firms; or 4.034 firms locates at the North and 9.232 ones at the South of Vietnam). The employed dataset is unbalanced panel with 13.266 observations, with descriptive statistics in Table 2.

**Table 2:** Descriptive Statistics of Private Firms. Source: Own analysis.

Variable	Obs.	Mean	Std. Dev.	Min	Max
Number of computers	13,266	0.828	2.431	0.000	100.000
*Value added*	13,266	5.242	1.204	1.946	8.001
Labour productivity	13,266	5.812	1.535	2.515	9.085
Capital intensive	13,266	3.913	1.082	1.166	6.393
Return on investment	13,266	0.066	1.194	−4.850	5.236
Return on Sales	13,266	0.033	0.173	−0.327	0.890
Leverage	13,266	0.296	0.261	0.000	0.915
Debt per Owner Equity	13,266	0.832	1.541	0.000	9.033
Start-up total assets	13,266	5.034	1.421	1.504	8.003
Start-up Leverage	13,266	0.218	0.213	0.000	0.835
Market concentration	13,266	0.024	0.046	0.001	0.301
Market share	13,266	0.029	0.101	0.000	0.661

## 4 Empirical results and discussion

The dependent variable in this survival model is the hazard rate, therefore a positive (negative) coefficient expresses that the corresponding factor increases (decreases) the instantaneous hazard of firm failure, or in other words, decreasing (increasing) the survival probability of firms. Based on the results of the Schoenfeld test and the Wald test, the proportional hazard assumption was not rejected while the null that all parameters are zero is rejected. In addition, the assumption of no unobserved heterogeneity was not rejected then the controlling for unobserved heterogeneity has helped unbiased estimates of explanatory variables (Esteve-Pérez and Máñez Castillejo, 2008).

### 4.1 Knowledge impact for whole sample

This section investigates the impact of knowledge factors on firm survival under controlling firm and industrial factors (Table 3). Model (1) analyses only the effect of knowledge factors; Model (2) examines how this effect change after controlling the effects of *Profitability, start-up factors, and Sustainability*, while Model (3) evaluates how the effects are modified after controlling *industrial factors*.

**Table 3:** Effects of knowledge factors on the Failure of new SMEs for whole sample.

Dependent variable: Hazard rate	Knowledge factors	Added internal factors	Added Industrial factors
	(1)	(2)	(3)
***Knowledge factors***			
Number of computers	−0.2745∗∗∗	−0.2377∗∗∗	−0.2361∗∗∗
	(0.038)	(0.038)	(0.038)
Value added	−0.1463∗∗∗	−0.1103∗∗∗	−0.1081∗∗∗
	(0.027)	(0.029)	(0.030)
Labor productivity	−0.0817∗∗∗	−0.0730∗∗∗	−0.0621∗∗∗
	(0.017)	(0.018)	(0.019)
Capital intensive	−0.0002	0.0032	−0.0021
	(0.034)	(0.034)	(0.034)
Lag of Capital intensive	−0.0265	0.0217	0.0181
	(0.030)	(0.033)	(0.033)
***Profitability***			
Return on investment		−*0.0276*∗	−*0.0279*∗
		(0.016)	(0.016)
Return on Sales		−*0.5083*∗∗∗	−*0.5434*∗∗∗
		(0.100)	(0.100)
***Start-up factors***			
Start-up total Assets		−0.0726∗∗∗	-0.0615∗∗∗
		(0.022)	(0.023)
Start-up Leverage		0.2482∗	0.2494∗
		(0.134)	(0.135)
***Sustainability***			
Leverage		−0.3672∗∗	−0.3308∗
		(0.180)	(0.180)
Debt per Owner Equity		0.0016	−0.0036
		(0.035)	(0.035)
***Industrial factors***			
Herfindahl–Hirschman Index			1.1966∗∗∗
			(0.288)
Market share			0.0372
			(0.115)
Log-likelihood	−11100.66	−11076.59	−11068.50
P value (Wald test)	(0.000)∗∗∗	(0.000)∗∗∗	(0.000)∗∗∗
$\chi^{2}$ (Schoenfeld test)	11.43	13.07	14.45
N. of firms	3237.00	3237.00	3237.00
N. of events	1447.00	1447.00	1447.00
N. of observations	13266	13266	13266

Note: Asterisks (∗), (∗∗), (∗∗∗) present statistical significance levels at least at the 10%, 5%, 1%, respectively. Standard errors in parentheses. Model (1) analyzes coefficient of knowledge factors. Model (2) expresses effects of added internal factors. Model (3) presents impact of added industrial factors.

Generally, the impact of knowledge factors consistently supports SMEs survival in various context, answering for the first research hypothesis (*The knowledge improves the survival of Vietnamese private SMEs*). The most beneficial knowledge factor for SMEs is number of computers, proxy for IT application, with the strongest effect, coefficient is −0.2745 (in model 1, Table 3) and the second strong effect belongs to value—added factor. These findings are meaningful for Vietnam, as this country standstills at the low-value-added chain in production during more than three decades of reform from 1986, compared with other more developed ASEAN countries. In addition, most of SMEs still employ out-of-date technologies, thus the failure hazard of Vietnamese private SMEs could be addressed by applying more IT, such as computers, or in other words, by the more development of knowledge economy.

The third knowledge factor favourable for firm survival is labour productivity, highlighting the importance of human capacity in increasing private SMEs’ survival, which is similar to various empirical findings (Baily et al., 1992; Disney et al. 2003; and Esteve-Pérez and Máñez Castillejo, 2008).

In addition, these effects of knowledge factors slightly decrease after controlling profitability, start-up factors, sustainability, and industrial factors in model 2 and model 3 (Table 3). In other words, when inserting more *internal and external* factors, the effect magnitude of knowledge factors changes, which confirms the second research hypothesis (*The effects of the knowledge on Vietnamese private SMEs'survival depend on internal and external factors*).

Regarding the group of profitability, all factors are significant and favourable for SMEs survival, expressing the market-selection power. Markedly, return on investment significantly contributes to SMEs firm survival while capital intensive does not. *This suggests that in the development of knowledge economy, the survival is one not with higher investment but rather higher efficiency of investment*.

More interestingly, the strongest determinant of private SMEs’ survival is Return on Sales, with coefficient of −*0.5083* (in model 2, Table 3) and this effect becomes stronger under the context of industrial factors (in model 3, Table 3). It implies that private SMEs with higher return on sales develop more sustainably under the condition of higher market concentration.

For start-up factors, the results noticeably express that the higher start-up total Assets will support private SMEs with the higher probability of survival. The start-up total assets seem to be a part of firm capacity to confront the insolvency risk. This result is similar to Xiaohong et al. (2010) with findings that Chinese enterprises with higher capital have higher survival opportunity. In addition, the vulnerable time may be at the start-up time for firms with higher leverage level.

In terms of Sustainability factors, Leverage significantly helps firms to increase their survival, interestingly compared with the effect of start-up leverage (significant and positive). This suggests that making use successfully leverage requires business experience which the new firms usually lack of.

In terms of industrial factors, higher HHI, presenting market concentration, threatens the private SMEs with a higher risk of failure. The higher market concentration means the higher competition pressure for exiting and the higher risk of insolvency which are more harmful for SMEs with micro, small and medium sizes (see Table 3).

### 4.2 Knowledge factors for different firm sizes

This section investigates how the effects of knowledge factors change among different cohort of firm sizes. Based on Vietnam legal regulation, the medium sized firm is one with more than ten employees, otherwise a small one. Firm size based on the number of employees has the advantage of avoiding being affected by flexible inflation like financial measures, thus it facilitates comparison with other studies.

The empirical results confirm the third hypothesis “The effects of knowledge on Vietnamese private SMEs ‘survival depend on firm size". While the effects of knowledge factors on small firms’ survival generally are the same as on whole sample, the medium firms only positively depend on labour productivity (see Table 4). This suggests that the knowledge factor in term of human capital will benefit medium sized firms in Vietnam. Particularly, the leverage supports strongly medium sized firms' survival while leverage – related factors are insignificant for small ones. It may explain that leverage just benefit firms with sufficient scale which helps firms to make use that leverage and avoid insolvency (see Table 4).

**Table 4:** Comparison of determinants of New-SMEs Failure by sizes.

	(1)	(2)	(3)
	Whole sample	Medium	Small
***Knowledge factors***			
Number of computers	**−**0.2361∗∗∗	**−**0.0451	**−***0.3229*∗∗∗
	(0.038)	(0.046)	(0.051)
Value added	**−**0.1081∗∗∗	**−**0.1021	**−***0.0350*
	(0.030)	(0.079)	(0.036)
Labor productivity	**−**0.0621∗∗∗	**−***0.1516*∗∗∗	**−***0.0763*∗∗∗
	(0.019)	(0.057)	(0.021)
Capital intensive	**−**0.0021	0.0111	**−**0.0422
	(0.034)	(0.088)	(0.039)
Lag of Capital intensive	0.0181	0.0154	0.0003
	(0.033)	(0.082)	(0.037)
***Profitability***			
Return on Investment	**−**0.0279∗	0.0383	**−**0.0415∗∗
	(0.016)	(0.038)	(0.017)
Return on Sale	**−**0.5434∗∗∗	**−**0.2198	**−**0.4809∗∗∗
	(0.100)	(0.411)	(0.103)
***Start-up factors***			
Start-up total Assets	**−**0.0615∗∗∗	**−**0.0600	**−**0.0428∗
	(0.023)	(0.066)	(0.025)
Start-up Leverage	0.2494∗	0.3781	0.2159
	(0.135)	(0.330)	(0.148)
***Sustainability***			
Leverage	**−**0.3308∗	**−**0.8201∗	**−***0.2794*
	(0.180)	(0.430)	(0.204)
Debt per Owner Equity	**−**0.0036	0.0247	0.0135
	(0.035)	(0.069)	(0.042)
***Industrial factors***			
Herfindahl–Hirschman Index	1.1966∗∗∗	0.4415	1.2329∗∗∗
	(0.288)	(1.508)	(0.296)
Market share	0.0372	0.1241	0.0546
	(0.115)	(0.223)	(0.136)
Log-likelihood	**−**11068.50	**−**1367.22	**−**9076.92
P value (Wald test)	(0.000)∗∗∗	(0.000)∗∗∗	(0.000)∗∗∗
$\chi^{2}$ (Schoenfeld test)	14.45	11.60	13.89
N. of firms	3237.00	1110.00	2859.00
N. of events	1447.00	218.00	1229.00
N. of observations	13266	3119	10147

Note: Asterisks (∗), (∗∗), (∗∗∗) present statistical significance levels at least at the 10%, 5%, 1%, respectively. Standard errors in parentheses. Model (1) analyzes coefficient of knowledge factors for whole sample. Model (2) expresses effects of knowledge factors for Medium – sized firms. Model (3) presents impact of knowledge factors for small – sized firms.

### 4.3 Knowledge factors for different firm locations

This section compares effects of knowledge factors on private SMEs by different location, based on the fact that different geographical locations with different conditions of business and production, including transportation, production resources, co-operators, and characteristics of customers, etc. will support firm operation at different levels.

There are outstanding differences between Northern and Southern private SMEs. While the effects of knowledge factors on the Southern firms' survival generally are the same as whole sample, only labour productivity supports the Northern ones’ survival (see Table 5).

**Table 5:** Comparison of determinants of New-SMEs Failure.

Dependent variable: Hazard rate	(1)	(2)	(3)
	Whole sample	North	South
***Knowledge factors***			
Number of computers	**−**0.2361∗∗∗	**−**0.0727	**−***0.3192*∗∗∗
	(0.038)	(0.055)	(0.050)
Value added	**−**0.1081∗∗∗	**−**0.1765∗∗∗	**−**0.0836∗∗
	(0.030)	(0.053)	(0.036)
Labor productivity	**−**0.0621∗∗∗	**−**0.0656∗	**−**0.0651∗∗∗
	(0.019)	(0.038)	(0.022)
Capital intensive	**−**0.0021	**−**0.0626	0.0114
	(0.034)	(0.068)	(0.039)
Lag of Capital intensive	0.0181	0.0087	0.0237
	(0.033)	(0.067)	(0.038)
***Profitability***			
Return on investment	**−**0.0279∗	**−**0.0075	**−**0.0347∗∗
	(0.016)	(0.036)	(0.017)
Return on Sales	**−**0.5434∗∗∗	**−**0.4527∗	**−**0.5233∗∗∗
	(0.100)	(0.260)	(0.109)
***Start-up factors***			
Start-up total Assets	**−**0.0615∗∗∗	**−**0.0346	**−**0.0658∗∗
	(0.023)	(0.046)	(0.026)
Start-up Leverage	0.2494∗	0.2222	*0.2480*
	(0.135)	(0.254)	(0.160)
***Sustainability***			
Leverage	**−**0.3308∗	**−**0.3720	**−***0.3098*
	(0.180)	(0.340)	(0.213)
Debt per Owner Equity	**−**0.0036	**−**0.0049	0.0044
	(0.035)	(0.062)	(0.043)
***Industrial factors***			
Herfindahl–Hirschman Index	1.1966∗∗∗	**−**1.2775	*1.3848*∗∗∗
	(0.288)	(1.209)	(0.302)
Market share	0.0372	0.1576	0.0125
	(0.115)	(0.197)	(0.141)
Log-likelihood	**−**11068.50	**−**2676.96	**−**7518.59
P value (Wald test)	(0.000)∗∗∗	(0.000)∗∗∗	(0.000)∗∗∗
$\chi^{2}$ (Schoenfeld test)	14.45	15.57	12.33
N. of firms	3237.00	968.00	2269.00
N. of events	1447.00	412.00	1035.00
N. of observations	13266	4034	9232

Note: Asterisks (∗), (∗∗), (∗∗∗) present statistical significance levels at least at the 10%, 5%, 1%, respectively. Standard errors in parentheses. Model (1) analyzes coefficient of knowledge factors for whole sample. Model (2) analyzes effects of knowledge factors for Northern firms. Model (3) expresses impact of knowledge factors for Southern firms.

Applying IT equipment like computer powerfully helps the Southern private SMEs’ survival while it is insignificant for the Northern ones (see Table 5). The reasons might be that the North includes the poorest region in the country, the northern mountains, especially North West where has a significantly strong negative effect on technology adaption of firms (Wider, 2011).

Market concentration becomes really harmful for the Southern but not for the Northern. The reasons might be the fact that firms located in the Northern regions are often more likely to engage in longer term contract arrangements (Wider, 2011), thus having better resilience capacity for market pressure. In addition, leverage – related factors turn to be insignificant for the Southern private SMEs (see Table 5).

## 5 Conclusions

Regarding the academic perspective, the paper contributes to the literature review on the performance of the enterprises in general and business performance of new private SMEs in the process of knowledge economy development in particular, and on the determinants of these firms' performance. This is a useful, highly reliable reference resource for researchers and policy consultants. From a practical perspective, the study's findings pave the way for solutions to adapt and capture opportunities in the knowledge economy. The research also contributes to the science base for supporting programs and policies, enhancing the competitiveness of enterprises, and for the policy of developing innovative eco-systems.

The findings are also sources of reference for researchers and training institutions, opening up a broader way of researching the performance of enterprises in the knowledge economy, and suggesting some policy solutions as follows:

First, the government should support firms in investing more in IT equipment, modern technology, creation and innovation, or R&D programs because the disadvantages of SMEs in developing countries commonly are the lack of capital and investment in creation and innovation. These investments are highly risky while their capital and capacity are limited at small and medium sizes. In addition, the implication for policy makers is to help these firms to improve labour productivity and value added by modernization, computerization and the development of human resources.

*Second,* there is evidence of market selection, based on *efficiency of investment.* Therefore, the government should facilitate investment, especially in knowledge, IT, human capacity. Besides, firms should improve *return on sales, especially in growing market.*

*Fourthly,* start-up SMEs should base on sufficient total assets, not on leverage to enter the market. And the government should support young firms with favourable financial source at beginning of firm business, and more easily accessible loans during its development process, especially for medium-sized firms.

*Finally*, regarding *external factors*, the results suggest policy makers should support firms in industries with high market concentration. This suggests policy makers should boost the economy restructure, and improve business environment to attract firms to enter high-market-concentration industries. Besides the government should have more financial supporting, such as favourable loans and rent, tax cuts, and the like, particularly when firms are small.

This research has some limitations which could be further researched. Firstly, the paper was only able to investigate number of computers, labour productivity, without total factor productivity (TFP) and total investment on R&D, innovation, human resource training, literacy rate and knowledge transfer due to the limitation of the data at the firm-level. In addition, further research should investigate the impact of present economic contexts, such as COVID-19 to Vietnamese SMEs.

## Declarations

### Author contribution statement

Nguyet Nguyen Thi, Conceived and designed the experiments; Performed the experiments; Analyzed and interpreted the data; Contributed reagents, materials, analysis tools or data; Wrote the paper.

### Funding statement

Assoc. Prof. Nguyet Nguyen Thi was supported by National Foundation for Science and Technology Development [502.01-2018.26, 2019-2021].

### Data availability statement

The authors do not have permission to share data.

### Declaration of interest’s statement

The authors declare no conflict of interest.

### Additional information

No additional information is available for this paper.
